# Supplementary material for: Combined Ammonia and Electron Processing of a Carbon-Rich Ruthenium Nanomaterial Fabricated by Electron-Induced Deposition
Source: Micromachines (Basel). 2020 Aug 12;11(8):769. doi: 10.3390/mi11080769 (PMC7466110; doi:10.3390/mi11080769)
Supplement: Supplementary file 1 [file micromachines-11-00769-s001.pdf]

Combined Ammonia and Electron Processing of a Carbon-Rich Ruthenium Nanomaterial Fabricated by Electron-Induced Deposition

Markus Rohdenburg, Johannes E. Fröch, Petra Martinović, Charlene J. Lobo and Petra Swiderek

AFM Data of as-Deposited Pads before NH_3 Treatment

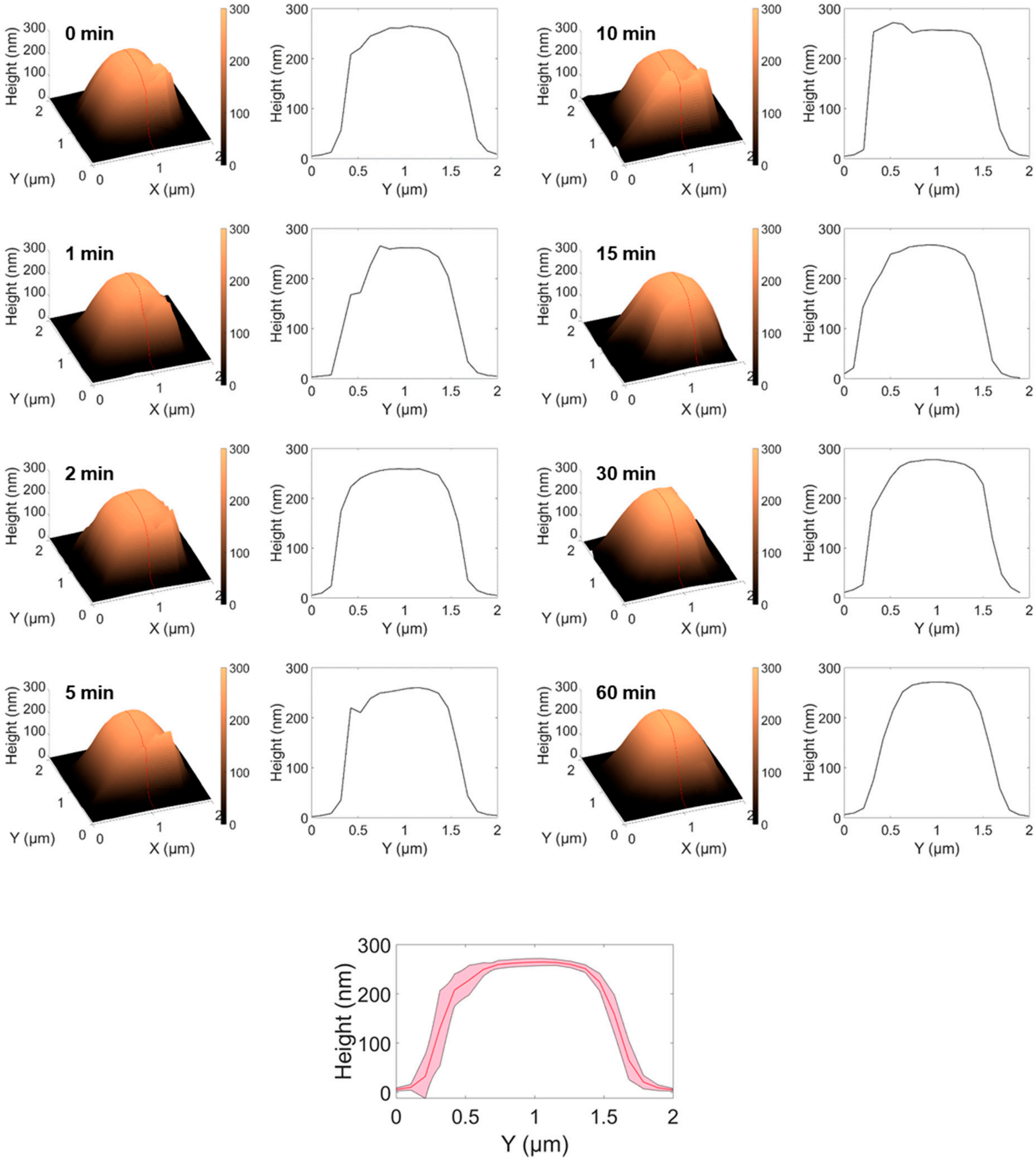

Figure S1. AFM images and cross sections of $(EtCp)_2Ru$ deposits on SiO_2/Si before treatment in an atmosphere of 0.11 mbar NH_3 by the 5 keV electron beam, with the times denoting the processing times used later in the experiment. The mean depth and standard deviation (shaded) are shown at the bottom.

AFM Data for Various Stages of NH_3 Treatment

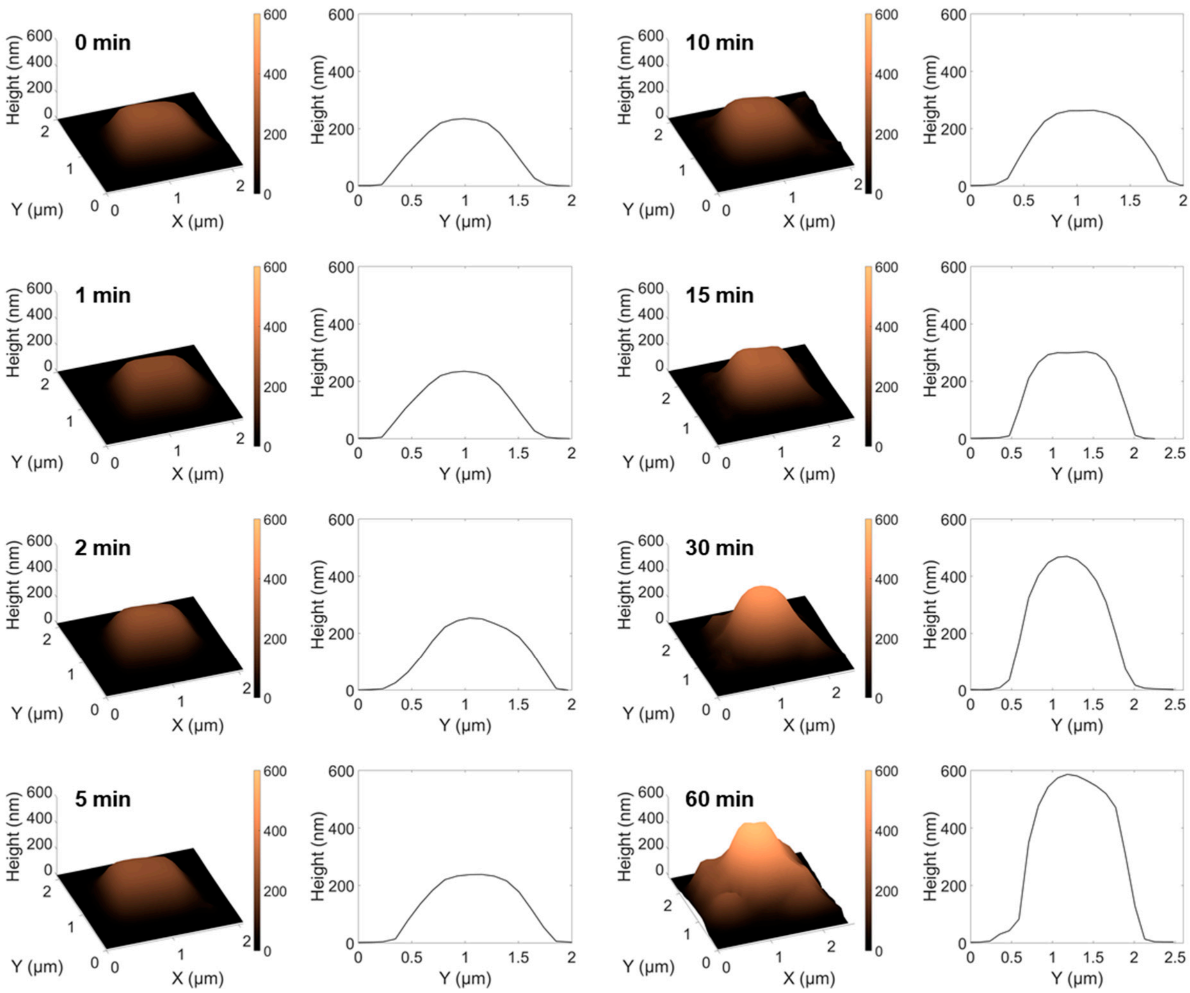

Figure S2. AFM images and cross sections of $(EtCp)_2Ru$ deposits on SiO_2/Si that have been treated in an atmosphere of 0.11 mbar NH_3 by the 5 keV electron beam for different processing times. Note the change in horizontal scale from 10 min to longer processing times.

Electron-Induced Reactions of NH_3 with the Si Substrate

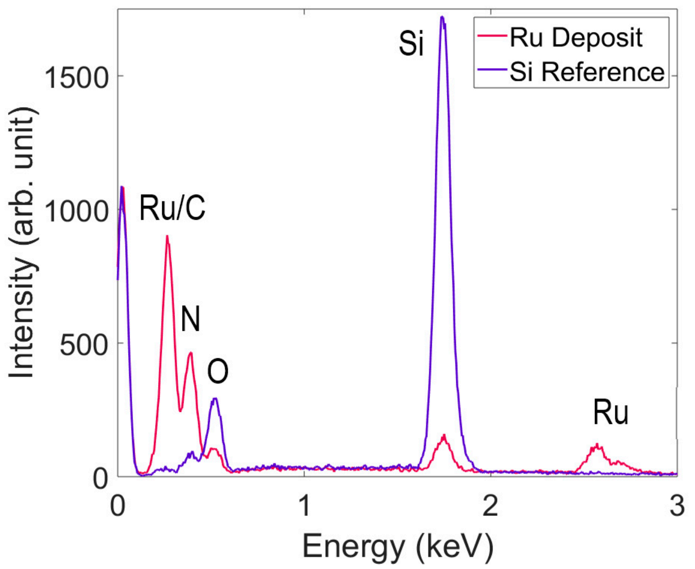

Figure S3. EDX spectra acquired after exposing the SiO_2/Si substrate in absence of a $(EtCp)_2Ru$ to a 5 keV electron beam at a beam current of 10 nA in an atmosphere of 0.11 mbar NH_3 for 60 min (purple) and for a $(EtCp)_2Ru$ deposit processed under the same conditions (red). The significantly increased N signal of the processed deposit indicates that electron-induced reactions of NH_3 with the Si substrate contribute only to a minor extent to the observed N incorporation into the deposits.

AFM Data for Various Stages of NH_3 Treatment of H_2O-Purified Deposits

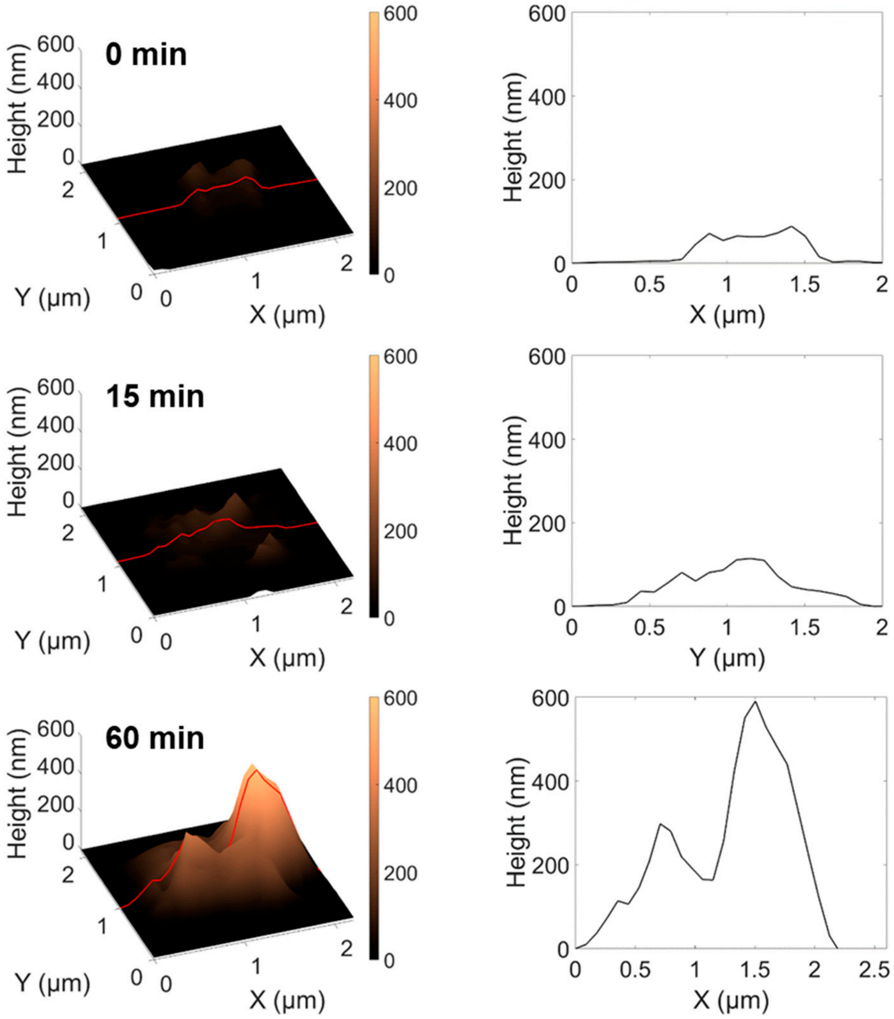

Figure S4. AFM images and cross sections of $(EtCp)_2Ru$ deposits on SiO_2/Si that have been treated in an atmosphere of 0.11 mbar NH_3 by the 5 keV electron beam for different processing times. The deposits had been pre-purified by water-assisted treatment before NH_3 treatment set in (5 keV, 0.13 mbar H_2O, 30 min).

ESD and TDS Data on Model Deposit Formation

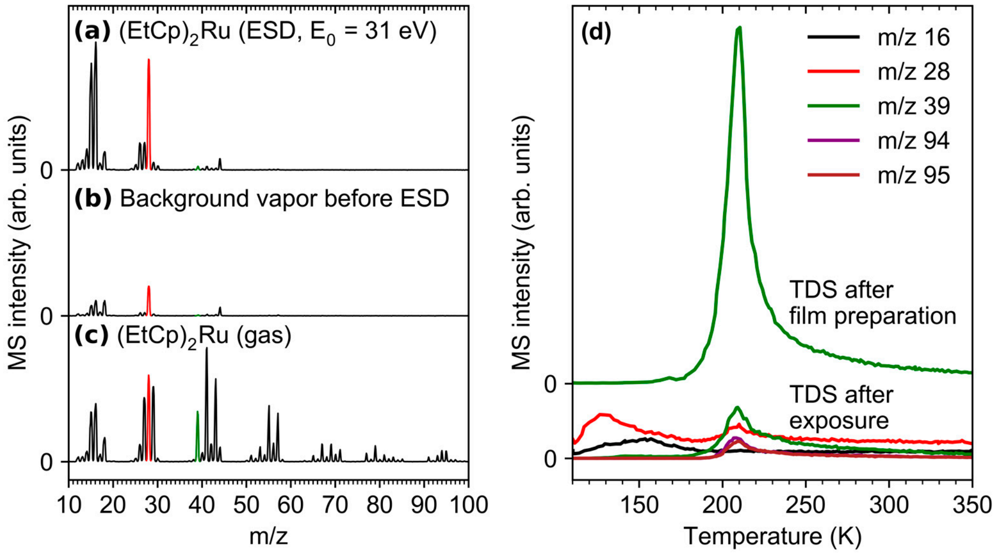

Figure S5. Mass spectra recorded (**a**) during electron exposure of 40 mC/cm² at E_0 = 31 eV of an adsorbed layer of $(EtCp)_2Ru$ with thickness of 13–20 ML on a Ta substrate held at 110 K, (**b**) before the start of irradiation, and (**c**) during dosing of $(EtCp)_2Ru$ onto the Ta substrate. The peak groups seen in ESD in the range m/z 12–16 give evidence of desorption of CH_4; m/z 26–30 reveal desorption of C_2H_4 and C_2H_6. Signals at m/z 39 and 41 are small, pointing to very little desorption of species related to the Cp ring [9]. (**d**) TDS experiments performed directly after preparation of the precursor layer and after electron exposure of 40 mC/cm² at E_0 = 31 eV. After the temperature ramp terminated at 350 K, the temperature was rapidly increased to 450 K where it was held for 30 s in a final annealing step. The figure is taken from reference [9] of the main manuscript. Reprinted with permission from Markus Rohdenburg, Robert Winkler, David Kuhness, Harald Plank, and Petra Swiderek, ACS Applied Nano Materials, DOI: 10.1021/acsanm.0c01759. Copyright 2020, American Chemical Society.

TDS After NH_3 Treatment

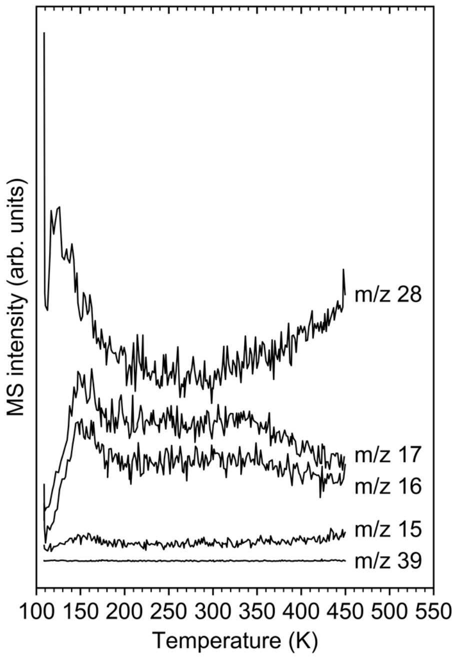

Figure S6. TDS of the volatile species present after ESD (E_0 = 31 eV, 40 mC/cm²) of an NH_3 film condensed on top of an $(EtCp)_2Ru$ model deposit.
